# Increased expression of transcription factor TFAP2α correlates with chemosensitivity in advanced bladder cancer

**DOI:** 10.1186/1471-2407-11-135

**Published:** 2011-04-14

**Authors:** Iver Nordentoft, Lars Dyrskjøt, Julie S Bødker, Peter J Wild, Arndt Hartmann, Simone Bertz, Jan Lehmann, Torben F Ørntoft, Karin Birkenkamp-Demtroder

**Affiliations:** 1 Molecular Diagnostic Laboratory, Department of Molecular Medicine, Aarhus University Hospital, Aarhus, Denmark; 2 Institute of Pathology, University Hospital Zürich, Zürich, Switzerland; 3 Department of Pathology, University of Erlangen, Erlangen, Germany; 4 Urology Practice Prüner Gang, Kiel, Germany

## Abstract

**Background:**

The standard treatment for patients with advanced transitional cell carcinoma of the bladder is platin based chemotherapy. Only approximately 50% of the patients respond to chemotherapy. Therefore, molecular predictive markers for identification of chemotherapy sensitive subgroups of patients are highly needed. We selected the transcription factor *TFAP2α *from a previously identified gene expression signature for chemotherapy response.

**Methods:**

*TFAP2α *expression and localization was assessed by immunohistochemistry using a tissue microarray (TMA) containing 282 bladder cancer tumors from patients with locally advanced (pT2-T4_b _and N_1-3_) or metastatic (M_1_) disease. All patients had received cisplatin containing chemotherapy. Furthermore, QPCR analysis of three *TFAP2α *isoforms was performed on tumor specimens of advanced muscle invasive bladder cancers (T2-4). Using the bladder cell lines T24 and SW780 the relation of *TFAP2α *and cisplatin and gemcitabine sensitivity as well as cell proliferation was examined using siRNA directed *TFAP2α *knockdown.

**Results:**

TFAP2α protein expression was analyzed on a TMA with cores from 282 advanced bladder cancer tumors from patients treated with cisplatin based combinational chemotherapy. *TFAP2α *was identified as a strong independent predictive marker for a good response and survival after cisplatin-containing chemotherapy in patients with advanced bladder cancer. Strong TFAP2α nuclear and cytoplasmic staining predicted good response to chemotherapy in patients with lymph node metastasis, whereas weak TFAP2α nuclear staining predicted good response in patients without lymph node metastasis. In vitro studies showed that siRNA mediated knockdown of TFAP2α increased the proliferation of SW780 cells and rendered the cells less sensitive to cisplatin and gemcitabine. In contrast to that T24 bladder cells with mutated p53 showed to be more drug sensitive upon TFAP2α depletion.

**Conclusions:**

High levels of nuclear and cytoplasmic TFAP2α protein were a predictor of increased overall survival and progression free survival in patients with advanced bladder cancer treated with cisplatin based chemotherapy. TFAP2α knockdown increased the proliferation of the SW780 bladder cells and reduced cisplatin and gemcitabine induced cell death. The inverse effect was observed in the *TP53 *mutated T24 cell line where TFAP2α silencing augmented cisplatin and gemcitabine sensitivity and did not stimulate proliferation.

## Background

Bladder cancer is the fifth most common malignancy in Europe and the fourth most common malignancy in the United States. The most prevalent histological type is transitional cell carcinoma (TCC), which constitutes up to 95% of the malignancies of the bladder. About 30% of TCC's display a solid, invasive growth pattern, being either locally advanced (pT3a, pT3b, pT4 and/or pN1, pN2, pN3 M0) or metastatic (M_1_) at the time of diagnosis or at later visits [1]. The response rate to chemotherapy is only approximately 50% [2]. Presently, there are two standard chemotherapeutic regimens: MVAC (methotrexate, vinblastine, doxorubicin, and cisplatin) or GC (gemcitabine and cisplatin). Median survival is 14 to 15 months, and 5-year overall survival rate is between 13% and 15% [3]. Although the gemcitabine and cisplatin combination has a significantly better toxicity profile, both regimens still carries risk for significant toxicity and toxic deaths [4] and a substantial fraction of patients will suffer from adverse reactions without achieving any benefit. It is therefore of utmost importance to be able to discriminate between responders and non-responders for improved selection of patients to chemotherapy and to improve the individual patient's quality of life. A molecular signature for chemo-resistance, based on microarray profiling of cancer specimens has been described for locally advanced and/or metastatic bladder cancer [5]. *BIRC5 *(*survivin*) and *BSG *(*emmprin*) were validated as independent predictive markers for response and survival after cisplatin-containing chemotherapy by immunohistochemistry in an independent material of 124 patients with locally advanced (T_4b _and N_2-3_) or metastatic (M_1_) disease [5]. In the present study we investigated another interesting molecule from this gene expression signature, the transcription factor Activator Protein TFAP2α. TFAP2α belongs to the TFAP2 family of transcription factors that in humans and mice consist of five members, TFAP2α, TFAP2β, TFAP2γ, TFAP2δ and TFAP2ε. Orthologs show a similarity between 60 and 99% at the amino-acid level. The TFAP2 family is characterized by having a highly conserved helix-span-helix dimerization motif at the carboxyl terminus together with a central basic region and a less conserved proline/glutamine rich domain at the amino terminus. The helix-span-helix domain facilitates homo and heterodimerization between the TFAP2 members. Once dimerized, the helix-span-helix motif and the neighboring basic domain facilitate DNA binding and the N-terminal proline/glutamine-rich domain mediates transactivation. The TFAP2 family members participate in the regulation of many signaling pathways and are essential during embryogenesis and development. TFAP2 proteins participate in tumorigenesis through regulation of neoplasia associated genes such as P21, Rb, TP53, ERα, BCL2, cKIT, MMP-2, E-cadherin and c-myc (reviewed in [6,7]). TFAP2α knockout mice are not viable and have severe ventral body wall closure defects (thoracoabdominoschisis)[8]. Currently many studies have linked deregulated TFAP2α activity to malignant transformation. The TFAP2α gene locus at 6p22 is frequently lost in various cancers [9]. Lost or decreased TFAP2α expression has been identified in human cancers of the breast, colon, prostate, ovary and brain [10-15] suggesting *TFAP2α *to be a tumor suppressor gene. Immunohistochemical analysis demonstrated a correlation between decreased *TFAP2α *expression and advanced colon adenocarcinomas [11]. In breast cancer, low nuclear *TFAP2α *expression is associated with disease progression and elevated metastatic capability [12]. Furthermore, reduced *TFAP2α *expression predicted elevated risk of recurrent disease in breast cancer [16]. Moreover, re-expression of *TFAP2α *in metastatic melanoma cells decreased their tumorigenity and inhibited their metastatic potential in nude mice [17]. So far the importance of TFAP2α in transitional cell carcinomas of the bladder has not been described. In the present study, we showed that high levels of nuclear and cytoplasmic TFAP2α protein were associated with increased overall survival and progression free survival of patients with lymph node positive advanced bladder cancer after cisplatin based chemotherapy. Furthermore, we showed that siRNA mediated knockdown of TFAP2α stimulated proliferation of the SW780 bladder cell line along with decreased cisplatin and gemcitabine sensitivity.

## Methods

Archival formalin-fixed paraffin embedded (FFPE) tissues of cystectomy specimens were derived from a previously reported Phase III, multicenter randomized control trial of two different adjuvant chemotherapy regimens, AUO-AB 05/95 conducted by the Arbeitsgemeinschaft Urologische Onkologien (AUO) collaborative group of the Federal Republic of Germany [18,19]. The clinicopathological characteristics of all patients were reviewed by one surgical pathologist (A.H.). Tumor staging was performed according to the criteria of the International Union against Cancer (UICC). Representative haematoxylin- and eosin-stained (HE) slides of formalin-fixed and paraffin-embedded tissue blocks obtained at cystectomy where reviewed and the tumor area was marked. Tissue microarrays (TMA) where constructed by obtaining a 1.5 mm punch-biopsy from each tumor. TMA construction was done as described previously [20]. The TMAs were stained with HE and were reviewed for the presence of representative tumor tissue by one surgical pathologist. The study was approved by the local ethical committee of the University Regensburg.

### Expression Plasmids

Plasmids of the human *TFAP2α *isoform 2 GenBank[NM_001032280] and 3 GenBank [NM_001042425]: pcDNA3.1/V5-His-*TFAP2α v2 and *pcDNA3.1/V5-His-*TFAP2α v3 *were generated by PCR amplification (Expand High Fidelity PCR System (Roche)) of bladder cancer patient cDNA as template using the primer pairs: 5'-GCCACCATGTTAGTTCACAGTTTTTCAGCC-3', 5'-CTTTCTGTGCTTCTCCTCTTTGTCACTG-3' and 5'- GAGCCGCGATGTCCATACTTGCC -3', 5'-CTTTCTGTGCTTCTCCTCTTTGTCACTG-3' respectively and cloned into the pcDNA3.1/V5-His^©^TOPO^©^TA expression vector (Invitrogen) following manufactures instructions.*TFAP2α *isoform 1 GenBank[NM_003220] was generated by sub-cloning from pcDNA3.1/V5-His-*TFAP2α v3 *using primer pair 5'-CCACCATGCTTTGGAAATTGACGGATAATATCAAGTACGAGGACTG-CGAGGACCGTCAC-3' and 5'-CTTTCTGTGCTTCTCCTCTTTGTCACTG-3'. The PCR product was subsequently cloned as described above.

### Western blotting

Whole cell protein lysates from COS7, T24 and SW780 were collected using a cell scraper and lysed in RIPA buffer (50 mM TRIS pH 7.5, 150 mM NaCl, 1% NP40, and 0.5% Deoxycholic Acid, sodium salt) supplemented with complete protease inhibitor cocktail (Boehringer Mannheim). Protein concentration was determined using Bradford reagent (BioRad laboratories, HerculesCA, USA). A 25 μg sample was resolved by SDS-PAGE (12% Tris-HCl, Invitrogen). Proteins were transferred to PVDF membrane (Millipore) and blocked overnight with 3% w/v skimmed milk powder in PBS buffer supplemented with 0.05% Tween-20. Membranes were probed with primary anti-TFAP2α mouse IgG (Abcam, ab18112 1:500) and subsequently, secondary horseradish peroxidase-conjugaed goat anti-mouse (Dako Cytomation 1:5000). The immunoreactive bands were visualized using ECL plus (Amersham Biosciences) and UVP ChemiDoc-It, Imaging system (UVP Inc.)

### Cell Culture

Bladder cell lines T24 and SW780 were obtained from American Type Culture Collection (ATCC-LGC standards, Borås, Sweden), were re-authenticated via STR analysis using the Cell-ID-system (G9500, Promega, Nacka, Sweden), products were analysed on an Applied-Biosystems3130 Genetic Analyser. No mycoplasma contamination was detected using nested PCR-based mycoplasma detection. COS-7 cells (green monkey) were cultured in RPMI 1640 and T24 (P53 Y126*, HRAS G12V) and SW780 bladder cells in DMEM with L-glutamine (Gibco, Invitrogen Corporation, Carlsbad, CA, USA) supplemented with 10% fetal bovine serum (Gibco), 100 U/ml penicillin (Gibco) and 100 U/ml streptomycin (Gibco) and maintained in a humidified atmosphere at 37 °C and 5% CO_2_.

### Transient transfection

The day prior to transfection 5 × 10^5 ^COS-7, 6 × 10^5 ^T24, 6 × 10^5 ^SW780 cells in T25 culture flasks (5 ml culture medium), 28 × 10^3^T24, 42 × 10^3 ^SW780 cells in 24-well plates (500 μl culture medium), 4 × 10^3 ^T24, 6 × 10^3 ^SW780 cells in 96-well (100 μl culture medium). Plasmid transfections were performed using FuGENE 6 (Roche) and siRNA transfections were performed using Lipofectamin 2000 both following the manufacturer's instructions. TFAP2α specific siRNA was obtained from Ambion (ABI;Foster City, CA)(Cat# s14003). Non-targeting siRNA was obtained from Dharmacon as Non-TARGET #2 si*CONTROL *(Cat# D-001208-14-20).

### Isolation of RNA

T24 and SW780 RNA was isolated from 6-well and 24-well plates using RNeasy spin columns (Qiagen, Hilden, Germany), as recommended by the manufacturer. RNA was quantified by measuring absorbance at 260 and 280 nm.

### Cell Proliferation Assay

Cell proliferation was measured using the CyQUANT cell proliferation assay (Invitrogen) according to the manufacturer's instructions. Fluorescence measurements were made using a microplate reader (Labsystems Multiscan MCC/340) with excitation at 485 nm and emission detection at 530 nm.

### Cell viability assay

The viability of sub-confluent cells was analyzed by 3-(4,5-dimethylthiazole-2-yl)-2,5-diphenyltetrazolium bromide (MTT) reduction assay. The assay was performed in 96 well plates seeding 4000-6000 cell/well in 200 μl. After 24 h 100 μl culture medium was carefully removed and 25 μl MTT solution was added (1 g MTT (Sigma M5655) dissolved in 200 mL D-PBS.) and stored shielded from light 1.5 h at 37°C and then 100 μl solubilization (50% dimethylformamide, 20% SDS) buffer was added and left protected from light ON. Readout was done using a microplate reader (Labsystems Multiscan MCC/340) at 540 nm. Absorbance at 692 nm was used as reference.

### Real-time RT-PCR

One μg of total RNA was used for cDNA synthesis with random hexamer primers using the SuperScript^® ^II Reverse Transcriptase (Invitrogen Calsbad, CA, USA) according to the manufacturer's instructions. Total RNA at 10-50 ng per 10 uL of reaction mixture was used for measurement of the target mRNA. The real-time RT-PCR assay was performed using the ABI 7500 FAST machine (ABI;Foster City, CA). 10 ul real-time RT-PCR reactions consists of 5 ul 2× TaqMan^® ^FAST Universal Master Mix (P/N 43660783, ABI; Foster City, CA), 0.5 ul 20 × TaqMan^® ^Assay/probe (ABI;Foster City, CA)) and cDNA equivalent to 10-50 ng of total RNA in 4.5 ul H2O. Thermal FAST cycle program was: 20 s at 95°C followed by 40 cycles of 3 s at 95°C and 30 s at 60°C. Reactions were set up in triplicate for each sample, and gene expressions were normalized to eukaryotic *Ubiquitin C *using SYBR GREEN PCR Master Mix (Applied Biosystems) and primers 5'-GATTTGGGTCGCGGTTCTT-3', 5'-TGCCTTGACATTCTCGATGGT-3' [21]. All assays were carried out in 96-well format plates covered with optical adhesive cover (P/N 4346906 and P/N 4311971 ABI, Foster City, CA). We used the 2^-ΔΔCT ^method to calculate the relative gene expression. TaqMan^® ^Assays used were: Hs01029410_m1 (TFAP2*α *isoform 1,2 and 3); Hs00231461_m1 (TFAP2*α *isoform); Hs01033609_m1 (TFAP2*α *isoform).

### Growth curves via RT-CES system (RTCA)

The T24 or SW780 cells were harvested and transferred into 16-well DP or 96-well SP E-Plates of the RTCA system, which contain electrodes integrated into the bottom surfaces of each well that measure cell index based on impedance using the RT-CES system (ACEA Biosciences, San Diego, CA, USA. This technology has recently been acquired by Roche Applied Science and is being marketed under the name xcelligence System). Changes in cell status such as cell number, viability and adherence were monitored in real time and quantified by detecting sensor electrical impedance. Cell index correlates with the number of cells attached to the bottom of the plate. For the experiments reported here, the number of cells added to each well varied from 4000 to 6000 per well with a total volume of 200 μl of media.

### Immunohistochemistry

Immunohistochemical analysis of FFPE bladder tissue sections (4 μm) and tissue microarray sections were performed essentially as described in [22]. The primary antibody against TFAP2α was ab18112 (mouse monoclonal, dilution 1:40, Abcam). Core biopsies of the TMA were either evaluated according to cytoplasmic staining (weak or strong intensity) or nuclear staining (weak or strong intensity). The TMA's were scored blinded and independently by two observers. In case of disagreement, the core was reevaluated and consensus was reached.

### Statistical analysis

All data calculations were done using the STATA statistical software (version 10).

## Results

### Expression of the individual variants of TFAP2α in advanced bladder cancer specimens

**Figure 1:**
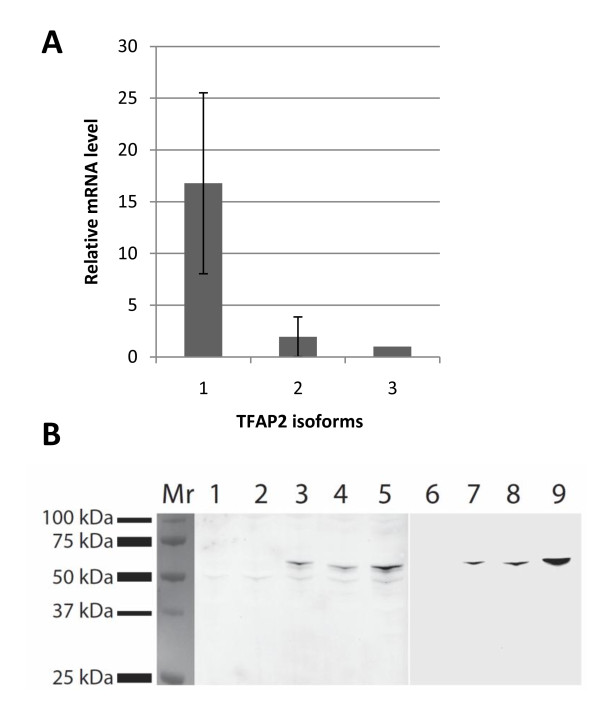
**Expression of TFAP2α isoforms**. (A) Expression of TFAP2α isoform 1, 2 and 3 in advanced muscle invasive bladder cancer (T2-4) were determined using real-time Q-PCR. Analysis was performed on cDNA from 10 tumor specimens and each bar represents the mean from the 10 samples.(B) COS-7 cells were transiently transfected with empty pcDNA3.1/V5-His vector (lane 2, 6), pcDNA3.1/V5-His-*TFAP2α *isoform 1(lane 3, 7), isoform 2 (lane 4, 8) and isoform 3 (lane 5, 9). Western blot of 30 μg total protein lysate from non-transfected HU609 bladder cells (lane 1) and COS-7 transfected cells (lane 2-9) 48 h post transfection probed with anti *TFAP2α *antibody (lane 1-5) or anti-V5 antibody (lane 6-9).

A real-time Q-PCR analysis was performed using cDNA from 10 tumor specimens of advanced muscle invasive bladder cancer (T2-4) to analyze the expression of the TFAP2α transcript variants. The analysis confirmed that all three variants of TFAP2α were present in all of the 10 tested bladder tumors (Figure 1A). The largest isoform TFAP2α-1 was most abundantly expressed compared to TFAP2α isoform 2 and 3. The mRNA level of TFAP2α isoform 1 was approximately 8 and 16 fold higher than isoform 2 and 3 respectively (p < 0.005). There is no statistical difference in expression levels between the mRNA level of isoform 2 and 3.

### TFAP2α expression in bladder carcinomas

**Figure 2:**
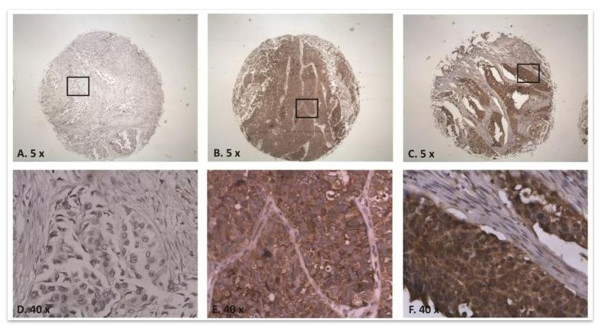
**Immunohistochemistry of bladder cancer tissue with antibody against *TFAP2α***. TFAP2α immunoreactivity of muscle invasive bladder cancer tumors. A and D: Negative stain of nucleus and cytoplasm at 5 and 40 fold objective magnification, respectively. B and E: Negative stain of nucleus and positive stain of cytoplasm (5× and 40×, respectively). C and F: Positive stain of nucleus and cytoplasm (5× and 40×, respectively).

**Figure 3:**
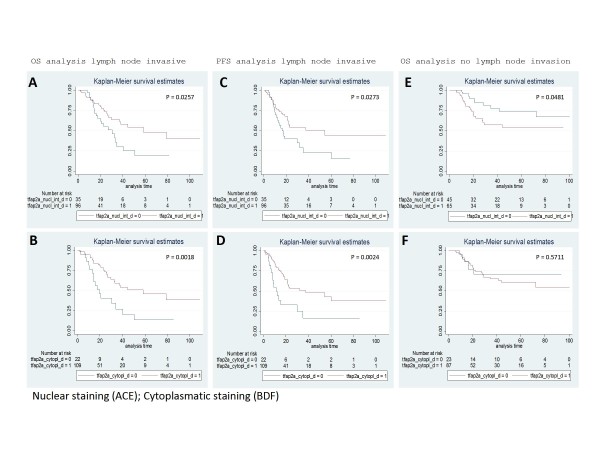
**The relationship between *TFAP2α *staining and survival after chemotherapy**. *TFAP2α *immunoreactivity and overall survival (OS) rates and progression free survival (PFS). A and B: separation of OS (overall survival) curves based on TFAP2α nuclear and cytoplasmic staining in the lymph node invasive group, respectively. C and D: separation of PFS curves based on TFAP2α nuclear and cytoplasmic staining in the lymph node invasive group, respectively. C and F: separation of OS curves based on TFAP2α nuclear and cytoplasmic staining in the non lymph node invasive group, respectively. Red curves are high TFAP2α staining and blue curves are low TFAP2α staining.

To assess the biological significance of *TFAP2α *expression in bladder tumors, we evaluated the expression using a TMA containing tumors from 282 patients with advanced bladder cancer with full clinical annotation (Table 1). The antibody specificity was tested successfully using western blots on total protein cell lysates (Figure 1B). Protein extracts of cell lysates from COS-7 cells transiently transfected with constructs containing one of the three different variants of *TFAP2*α isoforms 1-3 were used to evaluate the specificity of the antibodies against TFAP2α. The antibody detects all three isoform (lane 3-5) variants of the protein. The predicted MW of TFAP2α is 52 KDa in agreement with a slightly denser band detected due to the fusion of the V5 tag of the pcDNA3.1 cloning vector. The endogenous TFAP2α protein is seen below the fusion protein (lane 1-5). The TMA stained for TFAP2a yielded kappa values of (κ = 0.75 cytoplasmic expression) and (κ = 0.68 nucleic expression) between two observers, examples of typical staining patterns are shown in Figure 2. Kaplan-Meier survival analysis using all samples on the TMA showed no statistical association between overall survival or progression free survival, and cytoplasmic or nuclear TFAP2α staining. However, Kaplan-Meier survival analysis restricted to patient samples with lymph node invasion showed that both strong nuclear and cytoplasmic staining correlated with increased overall survival (p = 0.026 and p = 0.002 respectively(Figure 3A,B) and progression free survival (p = 0.027 and p = 0.002 respectively; Figure 3C,D). Interestingly, analysis of the samples without lymph node invasion showed an inverse correlation for the nuclear staining. Low nuclear TFAP2α staining was associated with increased overall survival rate (p = 0.048; Figure 3E). The cytoplasmic staining showed no correlation to outcome for the non lymph node invasive group (p = 0.57; Figure 3F).

**Table 1:** Patient Characteristics

Tumor category			
pTis7pT1 pN+	8(2.9)		
pT2 pN0	2(0.7)		
pT2 pN+	34(12.2)		
pT3 pN0	95(34.2)		
pT3 pN+	89(32.0)		
pT4a pN0	23(8.3)		
pT4a pN+	27(9.7)		
*			
**Age**	61.3(28.8-79.2)		
			
**Sex**			

Male	221(78)		
Female	61(22)		
			
**Nodal Status**			

pN0	124(44)		
pN+	158(56)		
			
**Survival parameters**		**Progression-free survival**	**Overall Survival**

pN0		32.9	34.9
pN+		21.5	24.5

*The stage was missing on four patients

### Cisplatin and Gemcitabine sensitivity of bladder Cancer cell lines

**Figure 4:**
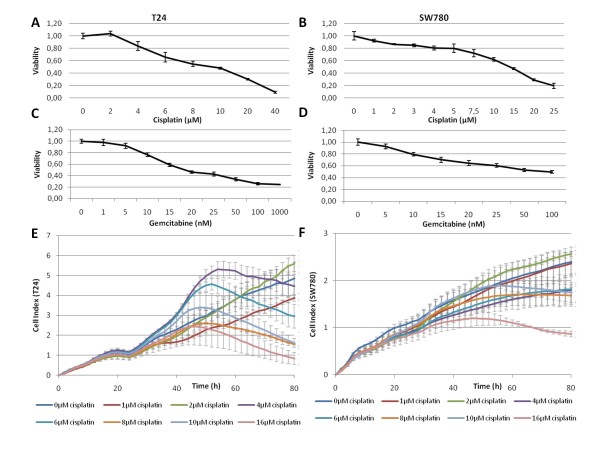
**Single agent dose-dependent cytotoxicity induced by cisplatin or gemcitabine**. Human bladder cancer cells T24 (A,C) and SW780 (B,D) were seeded in 96 well plates and treatment with the indicated cisplatin or gemcitabine concentration started 24 h after seeding and continued for 48 h. Cell viability was assessed with MTT-assay. Each drug concentration was tested in six individual wells. Real time growth curves monitoring was performed with the RT-CES (RTCA) system. The T24 or SW780 cells were seeded into 16-well or 96-well E-Plates, which contain electrodes integrated into the bottom surfaces of each well that measure cell index based on impedance. Cell index correlates with the area of cells attached to the bottom of the plate. Cisplatin (E) or gemcitabine (F) at the indicated concentration was added 24 h after seeding.

Studies of breast and colon cell lines show improved drug sensitivity of cells strongly expressing TFAP2α [11,12]. Furthermore, TFAP2α mediates its growth inhibition both directly through TP53 interaction and independently of TP53. In addition, TFAP2α binds directly to TP53 and stimulates p21 expression [23]. As our immunohistochemical analysis demonstrated decreased chemo sensitivity of bladder cancer patients with lymph node invasion having low *TFAP2α *expression we investigated if TFAP2α silencing would render bladder cancer cells less prone to chemotherapy drugs. The *TP53 *wild type bladder cancer cell line SW780 and the *TP53 *mutated bladder cancer cell line T24 were chosen to investigate the role of TFAP2α in chemo sensitivity. IC50 values of T24 and SW780 upon Cisplatin and Gemcitabine treatment were determined by an MTT viability assay (Figure 4ABCD). For Cisplatin the IC50(48 h) was around 10 μM for T24 and 15 μM for SW780. Gemcitabine had an IC50(48 h) of 15 μM for T24 and 50 μM for SW780. To verify the drug response we monitored the effect on cell proliferation in real time during drug incubation (Figure 4). Cisplatin was added after 24 h affecting the cells in a time period between 40-60 hours post-addition. For the T24 cells cisplatin concentration of 4 μM or more inhibit growth (the max cell index decreased, Figure 4E). At 72 h that corresponded to the MTT measurement the cell index of the 10 μM growth curve is 2.1 compared to 4.4 for the control curve without cisplatin. This approximately 50% reduction showed that the decreased surface area of the T24 cell population roughly corresponded to the viability readout. SW780 did not show the same association, but in agreement with the viability assay cisplatin inhibited cell growth substantially from 8 μM and 16 μM decreased growth heavily within the chosen 72 time window (Figure 4F).

### TFAP2α depletion decreased chemotherapy sensitivity in TP53 WT cells

**Figure 5:**
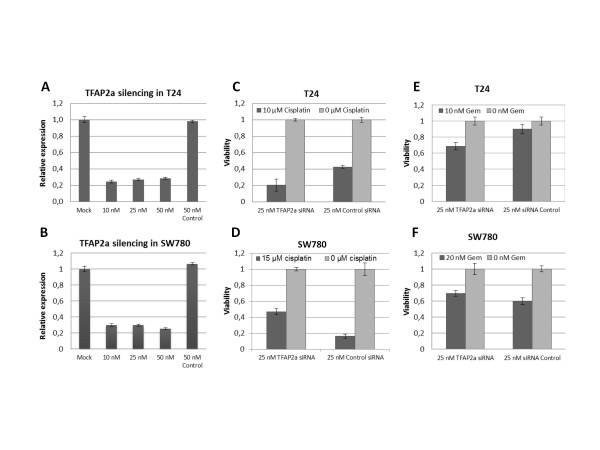
**Cisplatin sensitivity of TFAP2α silenced T24 and SW780**. A and B: Transfection of 10-50 nM TFAP2α siRNA or control siRNA in T24 and SW780 cells, respectively. Real-time RT-PCR was used to determine the relative TFAP2α mRNA levels 48 h post transfektion. C and D: Transfection of 25 nM TFAP2α siRNA in T24 and SW780 cells, respectively. After 24 h incubation cisplatin or media was added to the cells. The viability of the cells was determined 96 h after transfection (48 h after the drug was added) by MTT-assay and expressed as the viability compared with the culture media control for both the TFAP2α siRNA or control siRNA transfected cells. E and F: As C and D using gemcitabine instead of cisplatin. (n = 6).

T24 and SW780 cells were transfected with siRNA targeting TFAP2α transcript, yielding a 70% knock down 48 h post transfection (Figure 5AB). In order to perform the drug incubation without the cells getting too confluent the T24 and SW780 cells were first transfected in T25 culture flask and subsequently after 48 h incubation the cells were harvested and reseeded in 96 well plates. This extended time period did not have an impact on the knock down (data not shown). Next we added cisplatinor gemcitabine and incubated for 48 h followed by viability analysis (Figure 5C-F). TFAP2α silencing rendered SW780 less sensitive against cisplatin and gemcitabine induced cell death (Figure 5D and 5F), however in T24 cells the opposite effect was observed (Figure 5C and 5E).

### TFAP2α affected cell proliferation

**Figure 6:**
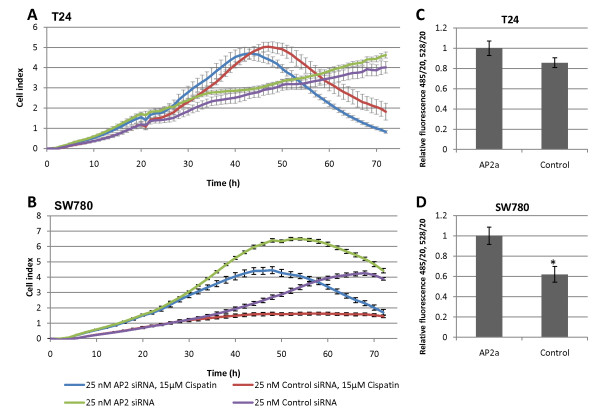
**Cell proliferation of TFAP2α silenced T24 and SW780**. Real time growth curves monitoring was performed with the RT-CES system. The T24 or SW780 cells were seeded into 16-well or 96-well E-Plates, which contain electrodes integrated into the bottom surfaces of each well that measure cell index based on impedance. Cell index correlates with the area of cells attached to the bottom of the plate. A and B: Transfection of 25 nM TFAP2α siRNA in T24 and SW780 cells, respectively. (n = 3) After 24 h incubation cisplatin or media was added to the cells. C and D: Transfection of 25 nM TFAP2α siRNA in T24 and SW780 cells, respectively. After 48 h incubation CyQuant assay was performed. (n = 8)

Visual inspection of the TFAP2α siRNA transfected SW780 cells showed a higher cell population compared to the SW780 cells transfected with siRNA control. To investigate the effect of TFAP2α siRNA knockdown on proliferation we performed real time proliferation analysis (Figure 6A and 5B). The real time proliferation monitoring of T24 showed no difference between the TFAP2α siRNA transfected and the siRNA control (Figure 6A) and the addition of cisplatin after 24 h did not change the proliferation rate either. The TFAP2α siRNA transfected SW780 cells however were significant proliferating faster than the siRNA control (Figure 6B). Addition of cisplatin after 24 h decreased the proliferation rate, especially for the TFAP2α siRNA knockdown. In conclusion TFAP2α downregulation stimulated proliferation of SW780 cell but not proliferation of T24 cells. Proliferation studies using a CyQuant proliferation assay confirmed these results (Figure 6C and 6D).

## Discussion

Studies investigating TFAP2α expression in human cancers show a correlation between reduced nuclear expression of TFAP2α and shorter recurrence-free survival and aggressive clinicopathological features in colon cancer [11], breast cancer [16], ovarian cancer [13] and melanoma [24]. Several *in vitro *studies demonstrated that TFAP2α has an inhibitory effect on cell proliferation and tumorigenesis and proposed that TFAP2α has a tumor suppressive effect in malignancies, although articles with opposing conclusions also have been published [7]. A large breast cancer study with immunohistochemical detection of TFAP2α revealed that a reduced level of TFAP2α in the nucleus and/or a shift of the protein to the cytoplasm may predict a shorter recurrence-free survival and breast cancer-related survival [7]. The observed correlation between high nuclear TFAP2α staining and decreased overall survival rate for the non lymph node invasive subgroup is in agreement with a breast cancer study from Finland that demonstrated that particular for lymph node positive patients low nuclear TFAP2α expression is associated with disease progression and elevated metastatic capability [25]. Reduced TFAP2α expression also predicted elevated risk of recurrent disease in breast cancer [16]. A study of human melanomas showed that high level of TFAP2α expression in the cytoplasm relative to the nucleus correlates with poor prognosis. The loss of nuclear TFAP2α expression was associated with malignant transformation and progression of melanoma, indicating that translocation of TFAP2α from the cytoplasm to the nucleus may be disrupted during melanoma progression. Thus it seems that the major deficiency in TFAP2α activity in metastatic melanoma is the loss of nuclear translocation. This could be due to modifications in the nuclear-pore complexes or in the activity of the transport receptors (karyopherines/importins/exportins) or changes of the TFAP2α protein itself. In our study, both low nuclear and cytoplasmic expression predicted poor outcome, suggesting that decrease of TFAP2α transcription/translation or increased turnover rate are a more likely course than translocation in the case of bladder cancer. For the group of patients without lymph node invasion the association was shifted, high nuclear staining was associated with decreased survival time. Although the correlation is not highly significant the difference from the lymph node invasive group is striking. There is no other study analyzing the TFAP2α staining in an isolated group of patients without lymph node invasion. The results of the TFAP2α staining analysis raised the question if the decreased chemo sensitivity of bladder cancer patients with lymph node invasion having low TFAP2α staining is due to depletion of TFAP2α or deregulation of a downstream target. We performed knock down experiments in bladder cell lines and subsequently measured their sensitivity against cisplatin and gemcitabine. Previous studies have shown that the tumor suppressor activity of TFAP2α is mediated through a direct interaction with *TP53*. Furthermore, TFAP2α induces TP53 dependent *p21 *transcriptional activation. This supports the observed ability of TFAP2α to induce G1 and G2 cell cycle arrest in *TP53*^+/+ ^but not in *TP53*^-/- ^HCT116 colon cells [23]. In contrast to this, a study with breast cancer cell lines demonstrated that TFAP2α down regulation decreases chemosensitivity irrespective of their TP53 status [26]. In light of these observations, we decided to use a TP53 mutated and a TP53 wild type bladder cell line to conduct functional chemosensitivity studies. We chose the T24 (TP53 homozygous Y126*) and the SW780 TP53 wild type line, both having approximately the same dose response profile of cisplatin and gemcitabine making them appropriate to compare. We showed that TFAP2α silencing rendered SW780 less sensitive against cisplatin and gemcitabine induced cell death and potentiated the cell death of T24 cells. Moreover, we found that TFAP2α down regulation stimulated proliferation of SW780 cell and did not change the proliferation rate of T24 cells. The SW780 cell line metastasizes to regional lymph nodes in nude mice tumor transplants, corresponding to the clinical findings in lymph node positive patients [27,28]. In contrast, TFAP2α silencing augments cisplatin and gemcitabine sensitivity and did not stimulate proliferation in the TP53 mutated and non-tumorigenic T24 bladder cell line, corresponding to clinical findings in lymph node negative patients. As mammalian cell terminal differentiate they undergo cell cycle arrest exiting from the cell cycle. TFAP2α mediates its role as a differentiation associated transcription factor through positive regulation of p21 thereby negatively regulating the cell cycle. TFAP2α induces expression of p21. The p21 promoter contains a TFAP2α binding site located at -103 and -95 where TFAP2α binds directly and stimulate expression [29]. In addition TFAP2α targets the p21 promoter in the p53 binding region at -2250, however only in the presence of p53 in agreement with TFAP2α has been shown to bind P53 *in vivo *and *in vitro *[23,30]. Furthermore TFAP2α induces p21 dependent P53 expression corroborating the observed ability of TFAP2α to induce G1 and G2 cell cycle arrest [23,31]. The explanation for the aberrant chemosensitivity of TFAP2α silenced T24 and SW780 could therefore be due to deregulation of the p53/p21 pathway in T24. For SW780, TFAP2α knockdown may suppress p53/p21 activation because TFAP2α is a positive regulate of p53/p21 expression. This potentiates the effect of cisplatin and gemcitabine as well as relief part of the suppression mediated by p53/p21 on the cell cycle. Taken together, our findings in T24 and SW780 cells may suggest that TFAP2α down regulation in bladder cells decreased cisplatin and gemcitabine sensitivity in a p53/p21 dependent manner. This is in line with studies showing that knock down of the TFAP2α expression in breast cancer and colon cancer cell lines resulted in significant reduction in chemotherapy-induced apoptosis [26,32]. In non-small cell lung cancer, expression of p53 and p21(Waf1) in mediastinal lymph node specimens were significantly related to the response to platinum chemotherapy [33]. Moreover, overexpression of TFAP2α expression in a breast cancer cell line augmented increased chemosensitivity and induced endogenous TFAP2α protein levels in a posttranscriptional way [26]. Within our study, re-introduction of TFAP2α in T24 and SW780 was performed by transient and stable transfection, Transient transfection was very low (<20%) as monitored by QPCR/WB and the selected clones seem to loose TFAP2α as no increased in transcript was measured (QPCR) compared to the mock transfected. In the literature, re-introduction of TFAP2α into TFAP2α-negative SW480 colon cancer cells stimulates expression of E-cadherin and down regulation of the MMP-9 expression and leads to dramatic loss of cellular invasive potential *in vitro*. Interestingly, node positive colorectal cancers showed significant losses for p21 and E-cadherin compared to node negative cancer [34]. TFAP2α directly binds to the promoter of E-cadherin, where it has been previously reported to act as a transcriptional activator [35]. High E-cadherin expression has been reported to increase cisplatin and gemcitabine sensitivity in pancreatic cancer [36]. Furthermore stable transfectants expressing TFAP2α in the SW480 cell line significantly inhibited their growth in an orthotopic animal model [35]. Previous studies have also demonstrated that re-expression of TFAP2α in SW480 cells resulted in an inhibition of colony formation *in vitro *and upregulation of p21^*Waf1/Cip1*^[31].

## Conclusions

In this study we demonstrated that high levels of nuclear and cytoplasmic TFAP2α protein was a predictor of improved overall survival and progression free survival of patients with locally advanced bladder cancer undergoing cisplatin based chemotherapy treatment when focusing on the lymph node invasive subgroup. In contrast, high nuclear TFAP2α staining was associated with decreased overall survival rate for the patients without lymph node metastases. Moreover, siRNA directed knock down of TFAP2α stimulated proliferation of SW780 bladder cells and decreased their cisplatin and gemcitabine sensitivity. On the contrary, TFAP2α silencing potentiated cisplatin and gemcitabine sensitivity and did not stimulate proliferation in the TP53 mutated T24 bladder cell line. Future studies are needed to further validate the predictive potential of TFAP2α expression in bladder cancer. Currently, we are collecting bladder tumor samples (locally advanced T3-4, N1-3 and/or metastatic M1) from patients that have been treated with cisplatin based chemotherapy and which have been characterized according to the RECIST response criteria. This cohort will be used to evaluate if TFAP2α staining and expression are predictive for cisplatin response in addition to survival.

## Competing interests

The authors declare that they have no competing interests.

## Authors' contributions

IN designed experiments, performed experiments, interpreted results, drafted manuscript. JSB designed experiments, performed experiments. LDA conducted statistical analysis, critical revision to manuscript. PJW, AH, SB provided TMA, critical revision to manuscript. JL conducted the clinical trial and provided the clinical data, critical revision of the manuscript. TFØ designed experiments, interpreted results and critical revision to manuscript. KBD designed experiments, optimized IHC, analyzed TMAs, interpreted results, and critical revision to manuscript

All authors have read and approved the final manuscript.

## Pre-publication history

The pre-publication history for this paper can be accessed here:

http://www.biomedcentral.com/1471-2407/11/135/prepub
